# A pilot histomorphology and hemodynamic of vasculogenic mimicry in gallbladder carcinomas *in vivo *and *in vitro*

**DOI:** 10.1186/1756-9966-30-46

**Published:** 2011-04-29

**Authors:** Wei Sun, Yue Z Fan, Wen Z Zhang, Chun Y Ge

**Affiliations:** 1 Department of Surgery, Tongji Hospital, Tongji University School of Medicine, Shanghai, China

**Keywords:** Gallbladder neoplasm, vasculogenic mimicry, 3-dimensional matrix, Xenograft model, Histomorphology, Hemodynamic

## Abstract

**Background:**

Vasculogenic mimicry (VM), as a new blood supply for tumor growth and hematogenous metastases, has been recently described in highly aggressive human melanoma cells, etc. We previously reported VM in human gallbladder carcinomas and its clinical significance. In this study, we further studied histomorphology and hemodynamic of VM in gallbladder carcinomas *in vivo *and *in vitro*.

**Methods:**

The invasive potential of human gallbladder carcinoma cell lines GBC-SD and SGC-996 were identified by Transwell membrane. The vasculogenic-like network structures and the signal intensities i.e. hemodynamic in gallbladder carcinomas stimulated via the three-dimensional matrix of GBC-SD or SGC-996 cells *in vitro*, the nude mouse xenografts of GBC-SD or SGC-996 cells *in vivo *were observed by immunohistochemistry (H&E staining and CD_31_-PAS double staining), electron microscopy and micro-MRA with HAS-Gd-DTPA, respectively.

**Results:**

Highly aggressive GBC-SD or poorly aggressive SGC-996 cells preconditioned by highly aggressive GBC-SD cells could form patterned networks containing hollow matrix channels. 85.7% (6/7) of GBC-SD nude mouse xenografts existed the evidence of VM, 5.7% (17/300) channels contained red blood cells among these tumor cell-lined vasculatures. GBC-SD xenografts showed multiple high-intensity spots similar with the intensity observed at tumor marginal, a result consistent with pathological VM.

**Conclusions:**

VM existed in gallbladder carcinomas by both three-dimensional matrix of highly aggressive GBC-SD or poorly aggressive SGC-996 cells preconditioned by highly aggressive GBC-SD cells *in vitro *and GBC-SD nude mouse xenografts *in vivo*.

## Background

The formation of a microcirculation (blood supply) occurs via the traditionally recognized mechanisms of vasculogenesis (the differentiation of precursor cells to endothelial cells that develop de novo vascular networks) and angiogenesis (the sprouting of new vessels from preexisting vasculature in response to external chemical stimulation). Tumors require a blood supply for growth and hematogenous metastasis, and much attention has been focused on the role of angiogenesis [1]. Recently, the concept of "vasculogenic mimicry (VM)" was introduced to describe the unique ability of highly aggressive tumor cells, but not to poorly aggressive cells, to express endothelium and epithelium-associated genes, mimic endothelial cells, and form vascular channel-like which could convey blood plasma and red blood cells without the participation of endothelial cells (ECs) [2]. VM consists of three formations: the plasticity of malignant tumor cells, remodelling of the extracellular matrix (ECM), and the connection of the VM channels to the host microcirculation system [3-5]. Currently, two distinctive types of VM have been described, including tube (a PAS-positive pattern) and patterned matrix types [6]. VM, a secondary circulation system, has increasingly been recognized as an important form of vasculogenic structure in solid tumors [2]. A lot of approaches have suggested that these VM channels are thought to provide a mechanism of perfusion and dissemination route within the tumor that functions either independently of or, simultaneously with angiogenesis [7-11]. VM channels and periodic acid-Schiff-positive (PAS) patterns are also associated with a poor prognosis, worse survival and the highest risk of cancer recurrence for the patients with melanoma [2,12], cell renal cell carcinoma [13], breast cancer [14], ovarian carcinoma [15], hepatocellular carcinoma [16-18], laryngeal squamous cell carcinoma [19], glioblastomas [20], gastric adenocarcinoma [21] colorectal cancer [22] and gastrointestinal stromal carcinoma [23].

Gallbladder carcinoma (GBC) is the most common malignancy of the biliary tract and the fifth common malignant neoplasm of the digestive tract in western countries [24,25]. It is also the most common malignant lesion of the biliary tract, the sixth common malignant tumor of the digestive tract and the leading cause of cancer-related deaths in China and in Shanghai [26]. 5-year survival for the patients lies between 0% and 10% in most reported series [26,27]. The poor prognosis of GBC patients is related to diagnostic delay, low surgical excision rate, high local recurrence and distant metastasis, and biological behavior of the tumor. Therefore, it is an urgent task to reveal the precise special biological behavior of GBC development, and provide a novel perspective for anticancer therapeutics. We previously reported the existence of VM in human primary GBC specimens and its correction with the patient's poor prognosis [28]. In addition, the human primary gallbladder carcinoma cell lines SGC-996, isolated from the primary mastoid adenocarcinoma of the gallbladder obtained from a 61-year-old female patient in Tongji Hospital were successfully established by our groups in 2003, the doubling time of cell proliferation was 48 h. Furthermore, we found SGC-996 cells accorded with the general characteristic of the cell line *in vivo *and *in vitro*. Based on these results, we hypothesized that the two different tumor cell lines, including GBC-SD and SGC-996, can exhibit significant different invasive ability and possess discrepancy of VM channels formation.

In this study, we show evidence that VM exists in the three-dimensional matrixes of human GBC cell lines GBC-SD (highly aggressive) and SGC-996 (poorly aggressive, but when placed on the aggressive cell-preconditioned matrix) *in vitro*, and in the nude mouse xenografts of GBC-SD cells *in vivo*. Taken together, these results advance our present knowledge concerning the biological characteristic of primary GBC and provide the basis for new therapeutic intervention.

## Methods

### Cell culture

Two established human gallbladder carcinoma cell lines used in this study were GBC-SD (Shanghai Cell Biology Research Institute of Chinese Academy of Sciences, CAS, China) and SGC-996 (a generous gift from Dr. Yao-Qing Yang, Tumor Cell Biology Research Institute of Tongji University, China). These cells were maintained and propagated in Dulbecco's modified Eagle's media (DMEM, Gibco Company, USA) supplemented with 10% fetal bovine serum (FBS, Hangzhou Sijiqing Bioproducts, China) and 0.1% gentamicin sulfate (Gemini Bioproducts, Calabasas, Calif). Cells were maintained at log phase at 37°C with 5% carbon dioxide.

### Invasion assay in vitro

The 35-mm, 6-well Transwell membranes (Coster Company, USA) were used to measure the *in vitro *invasiveness of two tumor cells. Briefly, a polyester (PET) membrane with 8-μm pores was uniformity coated with a defined basement membrane matrix consisting of 50 μl Matrigel mixture which diluted with serum-free DMEM (2 volumes versus 1 volume) over night at 4°C and used as the intervening barrier to invasion. Upper wells of chamber were respectively filled with 1 ml serum-free DMEM containing 2 × 10^5^·ml^-1 ^tumor cells (GBC-SD or SGC-996 cells, n = 3), lower wells of chamber were filled with 3 ml serum-free DMEM containing 1 × MITO+ (Collaborative Biomedical, Bedford, MA). After 24 hr in a humidified incubator at 37°C with 5% carbon dioxide, cells that had invaded through the basement membrane were stained with H&E, and counted by light microscopy. Invasiveness was calculated as the number of cells that had successfully invaded through the matrix-coated membrane to the lower wells. Quantification was done by counting the number of cells in 5 independent microscopic fields at a 400-fold magnification. Experiments were performed in duplicate and repeated three times with consistent results.

### Network formation assay in vitro

Thick gel of rat-tail collagen typeⅠwas made by mixing together ice-cold gelation solution, seven volumes of rat-tail collagen typeⅠ (2.0 mg·ml^-1^, Sigma Company, Germany) were mixed with two volumes of 10 × concentrated DMEM and one volume of NaHCO_3 _(11.76 mg·ml^-1^). Then 50 μl cold thick gel of rat-tail collagenⅠand Matrigel (Becton Dickinson Company, USA) were respectively dropped into a sterilized 35 mm culture dish (one 18 × 18 mm^2 ^glass coverslips placed on the bottom of dish) and allowed to polymerize for 30 min at room temperature, then 30 min at 37°C in a humidified 5% carbon dioxide incubator. The 7.5 × 10^5 ^tumor cells were then seeded onto the gels and incubated at 37°C with 5% carbon dioxide and humidity. The cultures were maintained in DMEM supplemented with 10% FBS and 0.1% gentamicin sulfate. The culture medium was changed every 2 days. In addition, on the premise of different invasion of two kinds of tumor cells, for experiments designed to analyze the ability of poorly aggressive tumor cells to engage in VM when placed on a matrix preconditioned by the highly aggressive tumor cells, which were removed after three days with 20 mM NH_4_OH followed by three quick washes with distilled water, phosphate buffered saline (PBS), and then complete medium. Followed by this experimental protocol, the highly aggressive tumor cells were cultured on a matrix preconditioned by the poorly aggressive tumor cells to explore the changes of remodeling capabilities. For experiments designed to analyze the ability of the cells to engage in VM using phase contrast microscopy (Olympus IX70, Japan). The images were taken digitally using a Zeiss Televal invertal microscopy (Carl Zeiss, Inc., Thornwood, NY) and camera (Nickon, Japan) at the time indicated.

### Tumor Xenograft assay in vivo

All of procedures were performed on nude mice according to the official recommendations of Chinese Community Guidelines. BALB/C nu/nu mice, 4 weeks old and about 20 grams, the ratio of male and female was 1:1 in this study. All mice were provided by Shanghai Laboratory Animal Center, Chinese Academy of Sciences (SLACCAS) and housed in specific pathogen free (SPF) condition. A volume of 0.2 ml serum-free medium containing single-cell suspensions of GBC-SD and SGC-996 (7.5 × 10^6^·ml^-1^) were respectively injected subcutaneously into the right axilback of nu/nu mice. In addition, the maximum diameter (a) and minimum diameter (b) were measured with calipers two times each week. The tumor volume was calculated by the following formula: V (cm^3^) = ∏ab^2^/6. The present study was carried out with approval from Research Ethical Review Broad in Tongji University (Shanghai, China).

### Immunohistochemistry in vitro and in vivo

For H&E staining: 12 paraffin-embedded tissue specimens of tumor xenografts were deparaffinized, hydrated, and stained with H&E. Companion serial section were stained with double staining of CD31 and PAS.

For CD_31 _and PAS double staining: Briefly, 12 paraffin-embedded tissue specimens (5 μm thickness) of the tumor xenografts were mounted on slides and deparaffinized in three successive xylene baths for 5 min, then each section was hydrated in ethanol baths with different concentrations. They were air-dried; endogenous peroxide activity was blocked with 3% hydrogen peroxide for 10 min at room temperature. The slides were washed in PBS (pH7.4), then pretreated with citratc buffer (0.01 M citric acid, pH6.0) for twice 5 min each time at 100°C in a microwave oven, then the slides were allowed to cool at room temperature and washed in PBS again, the sections were incubated with mouse monoclonal anti-CD_31 _protein IgG (Neomarkers, USA, dilution: 1:50) at 4°C overnight. After being rinsed with PBS again, the sections were incubated with goat anti-mouse Envision Kit (Genetech, USA) for 40 min at 37°C followed by incubation with 3, 3-diaminobenzidine (DAB) chromogen for 5 min at room temperature and washing with distilled water, then the section were incubated with 0.5% PAS for 10 min in a dark chamber and washing with distilled water for 3 min, finally all of these sections were counterstained with hematoxylin. The Microvessel in marginal area of tumor xenografts was determined by light microscopy examination of CD_31_-stained sections at the site with the greatest number of capillaries and small venules. The average vessel count of five fields (×400) with the greatest neovascularization was regarded as the microvessel density (MVD).

After glass coverslips with samples of three-dimensional culture were taken out, the samples were fixed in 4% formalin for 2 hr followed by rinsing with 0.01 M PBS for 5 min. The cultures were respectively stained with H&E and PAS (without hematoxylin counterstain). The outcome of immunohistochemistry was observed under light microscope with ×10 and ×40 objectives (Olympus CH-2, Japan).

### Electron microscopy in vitro and in vivo

For transmission electron microscopy (TEM), fresh tumor xenograft tissues (0.5 mm^3^) were fixed in cold 2.5% glutaraldehyde in 0.1 mol·L^-1 ^of sodium cacodylate buffer and postfixed in a solution of 1% osmium tetroxide, dehydrated, and embedded in a standard fashion. The specimens were then embedded, sectioned, and stained by routine means for a JEOL-1230 TEM.

### Dynamic MRA with intravascular contrast agent for xenografts in vivo

On day 21, when all the tumors of xenografts had reached at least 1.0 cm in diameter, they were examined by dynamic micro-magnetic resonance angiography (micro-MRA), MRI is a 1.5 T superconductive magnet unit (Marconic Company, USA). Two kinds of tumor xenograft nude mice (n = 2, for each, 7 weeks old, 35 ± 3 grams), anesthetized with 2% nembutal (45 mg·kg^-1^) intraperitoneal injection and placed at the center of the coils, were respectively injected I.V. in the tail vein with human adult serum gadopentetic acid dimeglumine salt injection (HAS-Gd-DTPA, 0.50 mmol (Gd)·l^-1^, Mr = 60-100kD, 0.1 mmol (Gd)·kg^-1^, gift from Department of Radiology, Tongji Hospital of Tongji University, China) before sacrifice. Micro-MRA was performed to analyze hemodynamic in the VM (central tumor) and angiogenesis (marginal tumor) regions. The images were acquired before injection of the contrast agents and 2, 5, and 15 min after injection. Three regions of interest (ROI) in the central area and the marginal area of the xenografted tumors and counted time-coursed pixel numbers per mm^3^. Two experiments were performed on these three gated ROI. All of the data (n = 6) were obtained directly from the MRA analyzer and were expressed as the mean ± SD.

### Statistical analysis

All data were expressed as mean ± SD and performed using SAS version 9.0 software (SAS Institute Inc., Cary, NC, USA). Statistical analyses to determine significance were tested with the χ2 or Student-Newman-Keuls *t *tests. *P *< 0.05 was considered statistically significant.

## Results

### Invasive potential of GBC-SD and SGC-996 cells in vitro

**Figure 1:**
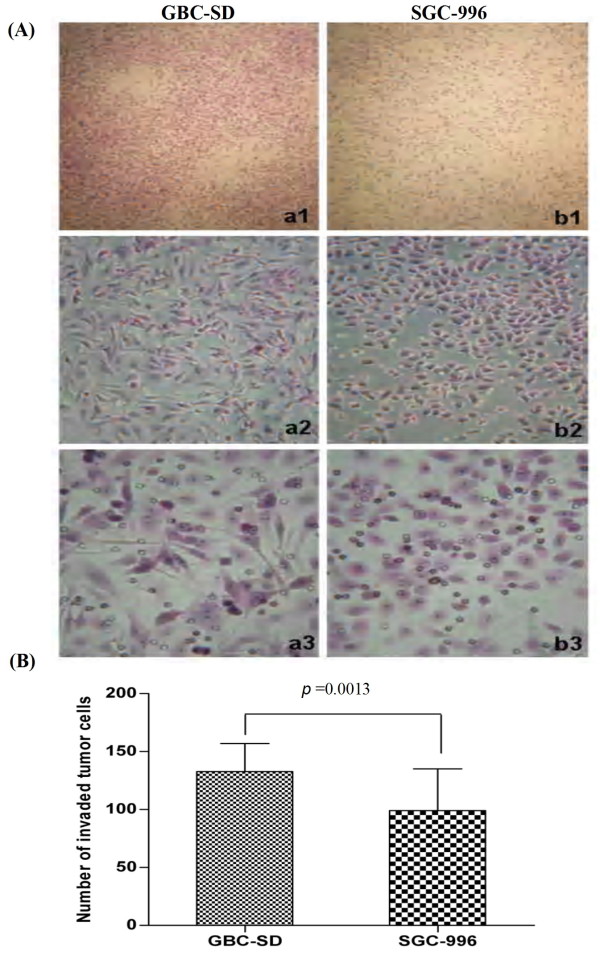
**Invasive potential of human gallbladder carcinoma cell lines GBC-SD and SGC-996 *in vitro***. **(A) **Representative phase contrast microscopy pictures of GBC-SD cells (***a***_***1-3***_; original magnification, ***a***_***1 ***_× 100, ***a***_***2 ***_× 200, ***a***_***3 ***_× 400) and SGC-996 cells (***b***_***1-3***_; original magnification, ***b***_***1 ***_× 100, ***b***_***2 ***_× 200, ***b***_***3 ***_× 400) with HE staining. Both GBC-SD and SGC-996 cells could invade through the matrix-coated membrane to the lower wells of Transwell plates. **(B) **The invaded number of GBC-SD cells were much more than that of SGC-996 cells (*P *= 0.0013).

The Transwell plates were used to measure the *in vitro *ability of cells to invade a basement membrane matrix--an important step in the metastatic cascade. We found the GBC-SD cells were mainly composed of spindle-shaped and polygonal cells. However, the SGC-996 cells could mainly form multi-layered colonies. The invasion results are summarized in Figure 1A. Both GBC-SD and SGC-996 cells could successfully invade through the matrix-coated membrane to the lower wells. However, the number of GBC-SD cells were much more than that of SGC-996 cells (137.81 ± 16.40 *vs*. 97.81 ± 37.66, *t *= 3.660, *P *= 0.0013). Hence, GBC-SD cells were defined as highly invasive cell lines, whereas SGC-996 cells were defined as poorly invasive cell lines (Figure 1B).

### Vessel-like structure formation in three-dimensional culture of GBC-SD and SGC-996 cells in vitro

**Figure 2:**
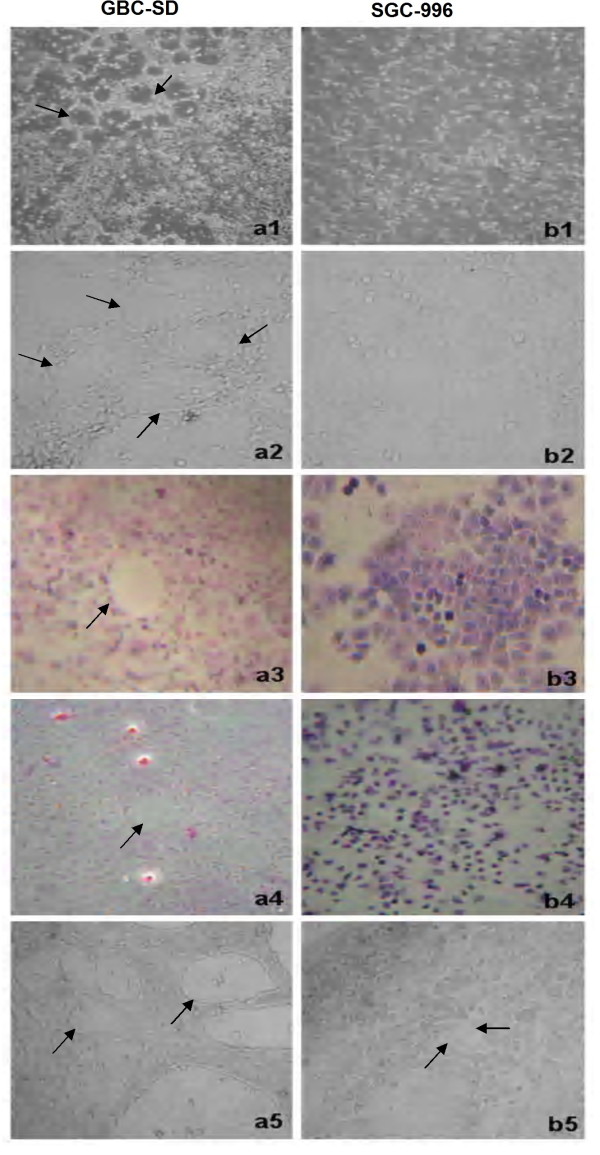
**Phase contrast microscopy of human gallbladder carcinoma cell lines GBC-SD (*a*) and SGC-996 (*b*) cultured three-dimensionally on Matrigel (*a***_***1***_**, *b***_***1***_**; original magnification × 100) and rat-tail collagenⅠmatrix (*a***_***2-5***_, ***b***_***2-5***_**, original magnification × 200) *in vitro***. Highly aggressive GBC-SD cells form patterned, vasculogenic-like networks when being cultured on Matrigel (***a***_***1***_) and rat-tail collagenⅠmatrix (***a***_***2***_) for 14 days. Similarly, the three-dimensional cultures of GBC-SD cells stained with H&E showed the vasculogenic-like structure at three weeks (***a***_***3***_); PAS positive, cherry-red materials found in granules and patches in the cytoplasm of GBC-SD cells appeared around the signal cell or cell clusters when stained with PAS without hematoxylin counterstain (***a***_***4***_). However, poorly aggressive SGC-996 cells did not form these networks when cultured under the same conditions (***b***_***1-4***_). GBC-SD cells cultured on a SGC-996 cells preconditioned matrix were not inhibited in the formation of the patterned networks by the poorly aggressive cell preconditioned matrix (***a***_***5***_). Poorly aggressive SGC-996 cells form pattern, vasculogenic-like networks when being cultured on a matrix preconditioned by the GBC-SD cells (***b***_***5***_).

As shown in Figure 2, highly aggressive gallbladder carcinoma GBC-SD cells were able to form network of hollow tubular structures when cultured on Matrigel and rat-tail collagen typeⅠcomposed of the ECM gel in the absence of endothelial cells and fibroblasts. The tumor-formed networks initiated formation within 48 hr after seeding the cells onto the matrix with optimal structure formation achieved by two weeks. Microscopic analysis demonstrated that the networks consisted of tubular structures surrounding cluster of tumor cells. During formation, the tubular networks became mature channelized or hollowed vasculogenic-like structure at two weeks after seeding the cells onto the gels. However, poorly aggressive SGC-996 cells were unable to form the tubular-like structures with the same conditions. After three days of incubation with the aggressive GBC-SD cells, these cells were removed, and poorly aggressive SGC-996 cells did assume a vasculogenic phenotype and initiated the formation of patterned, vessel-like networks when seeded onto a three-dimensional matrix preconditioned by aggressive GBC-SD cells (Figure 2b5). GBC-SD cells could still form hollowed vasculogenic-like structures when cultured on a matrix preconditioned by SGC-996 cells (Figure 2a5).

The three-dimensional cultures of GBC-SD cells stained with H&E showed the vasculogenic-like structure at two weeks (Figure 2a3). To address the role of the PAS positive materials in tubular networks formation, the three-dimensional cultures of GBC-SD cells were stained with PAS without hematoxylin counterstain. GBC-SD cells could secret PAS positive materials and the PAS positive materials appeared around the single cell or cell clusters. As an ingredient of the base-membrane of VM, PAS positive materials were located in granules and patches in the tumor cells cytoplasm (Figure 2a4). In contrast, the similar phenomenon didn't occur in SGC-996 cells (Figure 2b3, 2b4).

### VM's histomorphology of GBC-SD and SGC-996 xenografts in vivo

**Figure 3:**
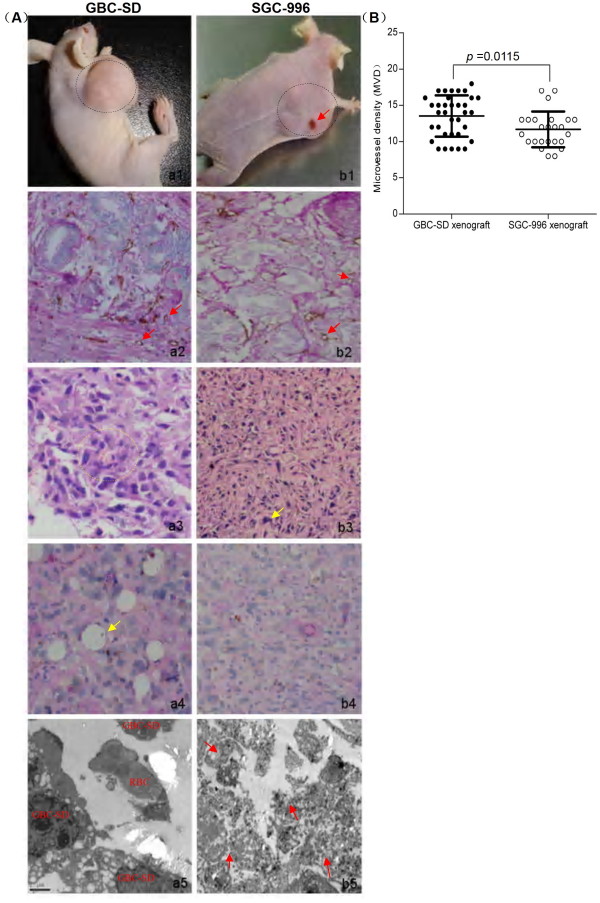
**Characteristic appearance and the histomorphologic observation of GBC-SD and SGC-996 xenografts *in vivo***. **(A) **GBC-SD (***a***_***1***_) and SGC-996 (***b***_***1***_) xenografts. Furthermore, SGC-996 xenografts exhibited different degree of tumor necrosis (***red arrowhead***). Immunohistochemistry with CD_31 _(original magnification × 200) revealed hypervascularity with a lining of ECs (***red arrowheads***), GBC-SD xenografts showed more angiogenesis in marginal area of tumor (***a***_***2***_) than that of SGC-996 xenografts (***b***_***2***_) [*P *= 0.0115, **(B)**]. Using H&E (***a***_***3***_***, b***_***3***_) and CD_31_-PAS double stain (***a***_***4***_***, b***_***4***_, original magnification × 200), sections of GBC-SD xenografts showed tumor cell-lined channels containing red blood cells (***a***_***3***_, ***yellow circle***) without any evidence of tumor necrosis. PAS-positive substances line the channel-like structures; Tumor cells form vessel-like structure with single red blood cell inside (***a***_***4***_, ***yellow arrowhead***). However, similar phenomenon failed to occur in SGC-996 xenografts (***b***_***3***_***, b***_***4***_) with tumor necrosis (***b***_***3***_, ***yellow arrowhead***). TEM (original magnification × 8000) clearly visualized several red blood cells in the central of tumor nests in GBC-SD xenografts (***a***_***5***_). Moreover, SGC-996 xenografts exhibited central tumor necrosis (***b***_***5***_, ***red arrowheads***) which consistent with morphology changes with H&E staining.

The tumor appeared gradually in subcutaneous area of right axilback of nude mice from the 6th day after inoculation. After 3 weeks, the tumor formation rates of nude mouse xenografts were 100% (7/7) for GBC-SD and 71.4% (5/7) for SGC-996 respectively. In addition, the medium tumor volume of GBC-SD xenografs was 2.95 ± 1.40 cm^3 ^(mean ± SD, range 1.73 to 4.86 cm^3^), while was 3.41 ± 0.56 cm^3 ^(mean ± SD, range 2.85 to 4.05 cm^3^) in SGC-996 xenografts, there was no significant difference between the two groups (Figure 3a1b1, P > 0.05).

H&E staining, dual-staining with CD_31_-PAS and TEM were used for xenografts to observe the morphology characteristic. Microscopically, in GBC-SD xenografts (n = 7, 4 μm-thick serial tissue specimens per nude mice model), the red blood cells were surrounded by tumor cell-lined channel and tumor cells presented various and obviously heteromorphism, necrosis was not observed in the center of the tumor (Figure 3a3a4). The channel consisted of tumor cells was negative of CD_31 _and positive PAS. Abundant microvessels appeared around the tumor, above all, in the marginal of the tumor. VM positive rate was 85.7% (6/7). Among 24 tissue sections, 10 high-power fields in each section were counted to estimate the proportion of vessels that were lined by tumor cells, 5.7% (17/300) channels were seen to contain red blood cells among these tumor cell-lined vasculatures. However, in SGC-996 xenografts (n = 5, 4 μm-thick serial tissue specimens per nude mice model), the phenomenon of tumor cell-lined channel containing the red blood cells were not discovered; the central area of tumor had the evidence of necrosis (Figure 3b3b4). In addition, in the marginal area of GBC-SD xenografts, hypervascularity with a lining of ECs was revealed, SGC-996 xenografts (Figure 3b2) exhibited less angiogenesis in the marginal area of the tumor than did GBC-SD (Figure 3a2). In the central area of tumor, GBC-SD xenografts exhibited VM in the absence of ECs, central necrosis, and fibrosis (Figure 3a3). Furthermore, the MVD of marginal area of tumor xenografts between GBC-SD and SGC-996 was compared. The MVD of GBC-SD xenografts (n = 7) was higher than the GBC-SD xenografts (n = 5, 13.514 ± 2.8328 *vs*. 11.68 ± 2.4617, *t *= 2.61, *P *= 0.0115) (Figure 3a2 b2).

For GBC-SD xenografts, TEM clearly showed single, double, and several red blood cells existed in the central of tumor nests. There was no vascular structure between the surrounding tumor cells and erythrocytes. Neither necrosis nor fibrosis was observed in the tumor nests (Figure 3a5). In contrast, the necrosis in GBC-SD xenografts specimens could be clearly found (Figure 3b5). These finding demonstrated that VM existed in GBC-SD xenografts and assumed the same morphology and structure characteristic as VM existed in human primary gallbladder carcinomas reported by us [28].

### Hemodynamic of VM and angiogenesis in GBC-SD and SGC-996 xenografts in vivo

**Figure 4:**
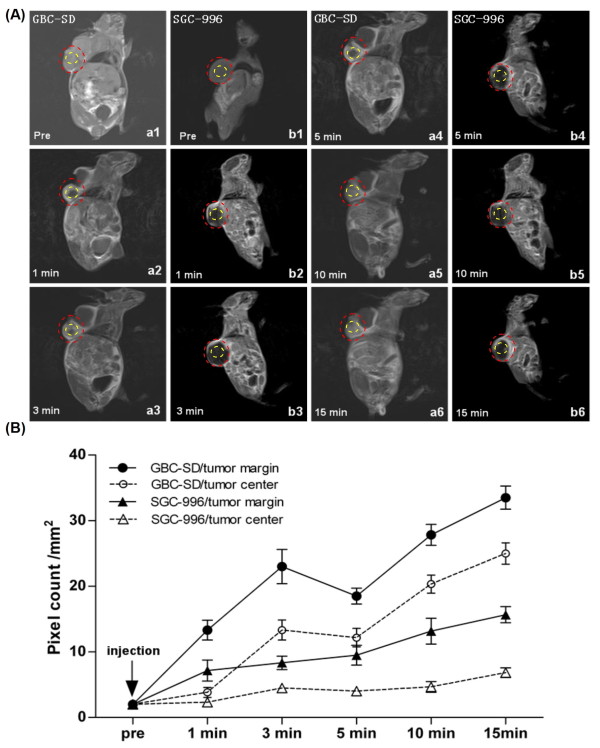
**Dynamic micro-MRA of the xenografts (*a***_***1-6***_**) and hemodynamic of VM and angiogenesis in GBC-SD and SGC-996 xenografts (*b***_***1-6***_**) *in vivo***. **(A) **The images were acquired before the injection of the contrast agents (HAS-Gd-DTPA, pre), 1, 3, 5, 10, and 15 min after injection. The tumor marginal area (***red circle***) of both GBC-SD and SGC-996 exhibited a signal that gradually increased in intensity. In the tumor center (***yellow circle***), GBC-SD exhibited spots in which the signal gradually increased in intensity (consistent with the intensity recorded for the tumor margin). However, the central region of SGC-996 maintained a lack of signal. **(B) **Hemodynamic of VM and angiogenesis in GBC-SD and SGC-996 nude mouse xenografts. All data are expressed as means ± SD. The time course of intensity of the tumor center (corresponding to the hemodynamic of VM) was consistent with the time course of intensity of tumor margin (corresponding to the hemodynamic of angiogenesis).

Two-mm-interval horizontal scanning of two different gallbladder carcinoma xenografts (GBC-SD and SGC-996) were conducted to compare tumor signal intensities between mice by dynamic Micro-MRA with an intravascular macromolecular MRI contrast agent named HAS-Gd-DTPA. As shown in Figure 4, the tumor marginal area of GBC-SD and SGC-996 xenografts exhibited gradually a high-intensity signal that completely surrounded the xenografted tumor, a finding consistent with angiogenesis. In the tumor center, GBC-SD xenografts exhibited multiple high-intensity spots (which is consistent with the intensity observed at tumor marginal), a result consistent with pathological VM. However, SGC-996 xenografts exhibited a low intensity signal or a lack of signal, a result consistent with central necrosis and disappearance of nuclei. Examination of the hemodynamic of VM revealed blood flow with two peaks of intensity and a statistically significant time lag relative to the hemodynamic of angiogenesis.

## Discussion

In the present study, we examined the capacity of GBC-SD and SGC-996 cell phenotypes and their invasive potential to participate in vessel-like structures formation *in vitro*, and succeeded in establishing GBC-SD and SGC-996 nude mouse xenograft models. In addition, highly invasive GBC-SD cells when grown in three-dimensional cultures containing Matrigel or typeⅠcollagen in the absence of endothelial cells and fibroblasts, and poorly aggressive SGC-996 cells when placed on the aggressive cell-preconditioned matrix could all form patterned networks containing hollow matrix channels. Furthermore, we identified the existence of VM in GBC-SD nude mouse xenografts by immunohistochemistry (H&E and CD31-PAS double-staining), electron microscopy and micro-MRA technique with HAS-Gd-DTPA. To our knowledge, this is the first study to report that VM not only exists in the three-dimensional matrixes of human gallbladder carcinoma cell lines GBC-SD *in vitro*, but also in the nude mouse xenografts of GBC-SD cells *in vivo*, which is consistent with our previous finding [28].

PAS-positive patterns are also associated with poor clinical outcome for the patients with melanoma [12] and cRCC [13]. In this study, we confirmed that VM, an intratumoral, tumor cell-lined, PAS-positive and patterned vasculogenic-like network, not only exists in the three-dimensional matrixes of human gallbladder carcinoma cell lines GBC-SD *in vitro*, but also in the nude mouse xenografts of GBC-SD cells *in vivo*. It is suggested that the PAS positive materials, secreted by GBC-SD cells, maybe be an important ingredients of base membrane of VM.

Tumor cell plasticity, which has also been demonstrated in prostatic carcinoma [29-31], bladder carcinoma [32], astrocytoma [33], breast cancer [34-38] and ovarian carcinoma [39-41], underlies VM. Consistent with a recent report, which show that poorly aggressive melanoma cells (MUM-2C) could form patterned, vasculogenic-like networks when cultured on a matrix preconditioned by the aggressive melanoma cells (MUM-2B). Furthermore, MUM-2B cells cultured on a MUM-2C preconditioned matrix were not inhibited in the formation of the patterned networks [42]. Our results showed that highly aggressive GBC-SD cells could form channelized or hollowed vasculogenic-like structure in three-dimensional matrix, whereas poorly aggressive SGC-996 cells failed to form these structures. Interestingly, the poorly aggressive SGC-996 cells acquired a vasculogenic phenotype and formed tubular vasculogenic-like networks in response to a metastatic microenvironment (preconditioned by highly aggressive GBC-SD cells). GBC-SD cells could still form hollowed vasculogenic-like structures when cultured on a matrix preconditioned by SGC-996 tumor cells. These data indicate that tumor matrix microenvironment plays a critical role in cancer progression. To date, several genes in tumor matrix microenvironment were revealed to participate in the process of VM and tumor cell plasticity. For example, over-expression of migration-inducing protein 7 (Mig-7) was found in aggressive invasive melanoma cells capable of VM but not in poorly invasive that do not form the tumor-lined structure. Over-expression of Mig-7 increased γ2 chain domain Ⅲ fragments known to contain epidermal growth factor (EGF)-like repeats that can activate EGF receptor. Laminin 5 is the only laminin that contains the γ2 chain, which following cleavage into promigratory fragments, the domain Ⅲ region, causes increased levels of matrix metalloproteinase-2 (MMP-2), and matrix metalloproteinase-14 (MMP-14) cooperate to cleave γ2 chain into fragments that promote melanoma cell invasion and VM [43,44]. However, in this study, we did not determine the molecular epigenetic effects induced by the matrix microenvironment preconditioned by highly aggressive GBC-SD cells. Molecular signal regulations of VM formation in GBC are supposed to be further studied. On the other hand, Sood *et al *[41] revealed the detailed scanning and transmission electron micrographs of ovarian cancer cell cultures grown on three-dimensional collagenⅠmatrices. The evident hollow tubular structures lined by flattened ovarian cancer cells could be observed by electron microscopy. In addition, they also found the tumor-formed networks initiated formation within 3 days after seeding the aggressive ovarian cancer cells onto the matrix. Furthermore, the tubular networks became channelized or hollowed during formation, and were stable through 6 weeks after seeding the cells onto a matrix, which is similar to our data, suggesting that hollow tubular structures might be the mature structures of VM when aggressive tumor cells were cultured on Matrigel or rat-tail collagen type Ⅰ.

VM, referred to as the "fluid-conducting-meshwork", may have significant implications for tumor perfusion and dissemination. Several papers evidenced the VM channel functional role in tumor circulation by microinjection method [3,7] and MRA technique [8,9,11]. We observed that VM only exists in GBC-SD xenografts by using H&E staining, CD_31_-PAS double staining and TEM, 5.7% channels were seen to contain red blood cells among these tumor cell-lined vasculatures, which is consistent with the ratio of human GBC samples (4.25%) [28]. We also found that GBC-SD xenografts exhibited much more microvessel in the marginal area of the tumor than did SGC-996 xenografts. In the central area of tumor, GBC-SD xenografts exhibited VM in the absence of ECs, central necrosis, and fibrosis. In contrast, SGC-996 xenografts exhibited central tumor necrosis as tumor grows in the absence of VM. This might suggest that the endothelial sprouting of new vessels from preexisting vessels as a result of over-expression of angiogenic factors. On the premise of successfully establishing GBC-SD and SGC-996 nude mouse xenografts, we furthermore performed dynamic micro-MRA analysis, using HAS-Gd-DTPA (60-100kD), which was much larger than Gd-DTPA (725D, generally MRI contrast agent) in molecule weight and volume. Thus the HAS-Gd-DTPA assumed much less leakage through the vascular wall than Gd-DTPA. Our results indicated that the hemodynamic of VM revealed blood flow with two peaks of intensity and a statistically significant time lag, relative to the hemodynamic of angiogenesis, which is consistent with the reported findings [9,11], suggesting that VM might play role in perfusion and dissemination of GBC-SD xenografted tumors as the fluid-conducting-meshwork. Taken together, these data also provided strong evidence the connection between angiogenesis and VM in GBC-SD xenografts.

## Conclusions

In conclusion, the present study reveals that VM exists in GBC by both three-dimensional matrix of highly aggressive GBC-SD or poorly aggressive SGC-996 cells preconditioned by highly aggressive GBC-SD cells *in vitro *and GBC-SD nude mouse xenografts *in vivo*. This study has a limitation that only two different established GBC cell lines in China were enrolled in present study. Hence, we couldn't draw a comprehensive conclusion about biological characteristic of GBC. However, our study provides the background for continuing study for VM as a potential target for anticancer therapy in human GBC. Therefore, furthermore studies are needed to clarify the molecular mechanism of VM in the development and progression of GBC.

## Abbreviations

VM: vasculogenic mimicry; ECs: endothelial cells; ECM: extracellular matrix; PAS: periodic acid-Schiff-positive; GBC: Gallbladder carcinoma; SPF: specific pathogen free; DMEM: Dulbecco's modified Eagle's media; FBS: fetal bovine serum; MVD: microvessel density; TEM: transmission electron microscopy; HAS-Gd-DTPA: human adult serum gadopentetic acid dimeglumine salt injection; ROI: regions of interest; Mig-7: migration-inducing protein 7; EGF: epidermal growth factor; MMP: matrix metalloproteinase.

## Competing interests

The authors declare that they have no competing interests.

## Authors' contributions

W Sun and YZ Fan were responsible for data collection and analysis, experiment job, interpretation of the results, and writing the manuscript. W Sun carried out the Invasion assay and three-dimensional culture of GBC-SD and SGC-996 cells *in vitro*. WZ Zhang and CY Ge carried out the nude mouse xenografts of GBC-SD and SGC-996 cells. W Sun and WZ Zhang were responsible for the existence of VM in GBC by using immunohistochemistry staining, TEM and micro-MRA technology *in vitro *and *in vivo*, respectively. All authors have read and approved the final manuscript.
